# Supplementary material for: Whole Exome-Wide Association Identifies Rare Variants in GALNT9 Associated with Middle Eastern Papillary Thyroid Carcinoma Risk
Source: Cancers (Basel). 2023 Aug 24;15(17):4235. doi: 10.3390/cancers15174235 (PMC10486701; doi:10.3390/cancers15174235)
Supplement: Supplementary file 1 [file cancers-15-04235-s001.zip › cancers-2538843-supplementary.pdf]

Supplementary Table S1: List of variants significantly related to PTC risk in seven SKAT genes

S.No.	Chr	Position	Ref	Alt	Gene	No. of Cases	%	No. of Controls	%	P-value
1.	chr6	31599982	CCT	-	PRRC2A	6	2.4	0	0.0	6.31E-09
2.	chr6	30114877	C	T	TRIM40	5	2.0	0	0.0	1.15E-07
3.	chr6	31759739	C	G	VARS1	4	1.6	0	0.0	2.14E-06
4.	chr6	31600297	G	A	PRRC2A	4	1.6	0	0.0	2.14E-06
5.	chr6	30075877	G	C	TRIM31	4	1.6	0	0.0	2.14E-06
6.	chr6	31746814	C	T	VARS1	3	1.2	0	0.0	4.07E-05
7.	chr6	31600670	G	A	PRRC2A	3	1.2	0	0.0	4.07E-05
8.	chr6	28547154	T	C	ZBED9	2	0.8	0	0.0	8.10E-04
9.	chr6	31595795	C	T	PRRC2A	2	0.8	0	0.0	8.10E-04
10.	chr6	31734385	C	T	VWA7	2	0.8	0	0.0	8.10E-04
11.	chr6	31737844	T	A	VWA7	2	0.8	0	0.0	8.10E-04
12.	chr6	30079443	G	T	TRIM31	2	0.8	0	0.0	8.10E-04
13.	chr1	36564812	G	A	COL8A2	2	0.8	0	0.0	8.10E-04
14.	chr6	31746847	C	T	VARS1	1	0.4	0	0.0	0.0179
15.	chr6	31746948	C	G	VARS1	1	0.4	0	0.0	0.0179
16.	chr6	31747903	G	A	VARS1	1	0.4	0	0.0	0.0179
17.	chr6	31750602	C	T	VARS1	1	0.4	0	0.0	0.0179
18.	chr6	28541333	C	T	ZBED9	1	0.4	0	0.0	0.0179
19.	chr6	28542480	G	T	ZBED9	1	0.4	0	0.0	0.0179
20.	chr6	28543044	G	A	ZBED9	1	0.4	0	0.0	0.0179
21.	chr6	28543091	G	T	ZBED9	1	0.4	0	0.0	0.0179
22.	chr6	28543496	TCTT	-	ZBED9	1	0.4	0	0.0	0.0179
23.	chr6	28554429	A	C	ZBED9	1	0.4	0	0.0	0.0179
24.	chr6	31598523	C	T	PRRC2A	1	0.4	0	0.0	0.0179
25.	chr6	31599131	C	T	PRRC2A	1	0.4	0	0.0	0.0179
26.	chr6	31599293	G	T	PRRC2A	1	0.4	0	0.0	0.0179
27.	chr6	31599370	G	C	PRRC2A	1	0.4	0	0.0	0.0179
28.	chr6	31600696	G	A	PRRC2A	1	0.4	0	0.0	0.0179
29.	chr6	31603498	A	G	PRRC2A	1	0.4	0	0.0	0.0179
30.	chr6	31604009	C	T	PRRC2A	1	0.4	0	0.0	0.0179
31.	chr6	31733701	G	A	VWA7	1	0.4	0	0.0	0.0179
32.	chr6	31734293	G	A	VWA7	1	0.4	0	0.0	0.0179
33.	chr6	31735240	C	T	VWA7	1	0.4	0	0.0	0.0179
34.	chr6	31736948	C	A	VWA7	1	0.4	0	0.0	0.0179
35.	chr6	31736974	-	CTCACC	VWA7	1	0.4	0	0.0	0.0179
36.	chr6	31741049	C	A	VWA7	1	0.4	0	0.0	0.0179
37.	chr6	31742385	G	A	VWA7	1	0.4	0	0.0	0.0179
38.	chr6	31743953	G	A	VWA7	1	0.4	0	0.0	0.0179
39.	chr6	30073004	T	A	TRIM31	1	0.4	0	0.0	0.0179
40.	chr6	30105113	G	C	TRIM40	1	0.4	0	0.0	0.0179
41.	chr6	30113787	G	A	TRIM40	1	0.4	0	0.0	0.0179

42.	chr1	36563964	C	A	COL8A2	1	0.4	0	0.0	0.0179
43.	chr1	36564493	C	A	COL8A2	1	0.4	0	0.0	0.0179
44.	chr1	36564587	CCAGGGAGGCC GGGGGGGCCGG GGGCACCCCCCT GCCCTGGGGCC	-	COL8A2	1	0.4	0	0.0	0.0179
45.	chr1	36565019	C	T	COL8A2	1	0.4	0	0.0	0.0179
46.	chr1	36565773	G	C	COL8A2	1	0.4	0	0.0	0.0179
